# Supplementary material for: Deletion of SERF2 in mice delays embryonic development and alters amyloid deposit structure in the brain
Source: Life Sci Alliance. 2023 May 2;6(7):e202201730. doi: 10.26508/lsa.202201730 (PMC10155860; doi:10.26508/lsa.202201730)
Supplement: Supplementary file 1 [file LSA-2022-01730_TableS1.docx]

| **Initial breeding schemes** | **Serf2**^+/+^ | **Serf2**^+/-^ | **Serf2**^-/-^ | **Unknown** | **Total** |
| --- | --- | --- | --- | --- | --- |
| ***Serf2*^-/-^ X *Serf2*^+/-^** | - | 32 | 1 | 1 | **34** |
| ***Serf2*^+/-^ X  *Serf2*^+/-^** | 27 | 64 | 0 | 5 | **96** |

**Table 1**: Number of first generation pups observed in the various genotype groups using 4 *Serf2*^-/-^ and 8 S*erf2*^+/-^ breeding animals.
