# Supplementary figures and images for: Deletion of SERF2 in mice delays embryonic development and alters amyloid deposit structure in the brain
Source: Life Sci Alliance. 2023 May 2;6(7):e202201730. doi: 10.26508/lsa.202201730 (PMC10155860; doi:10.26508/lsa.202201730)

Figure 1D

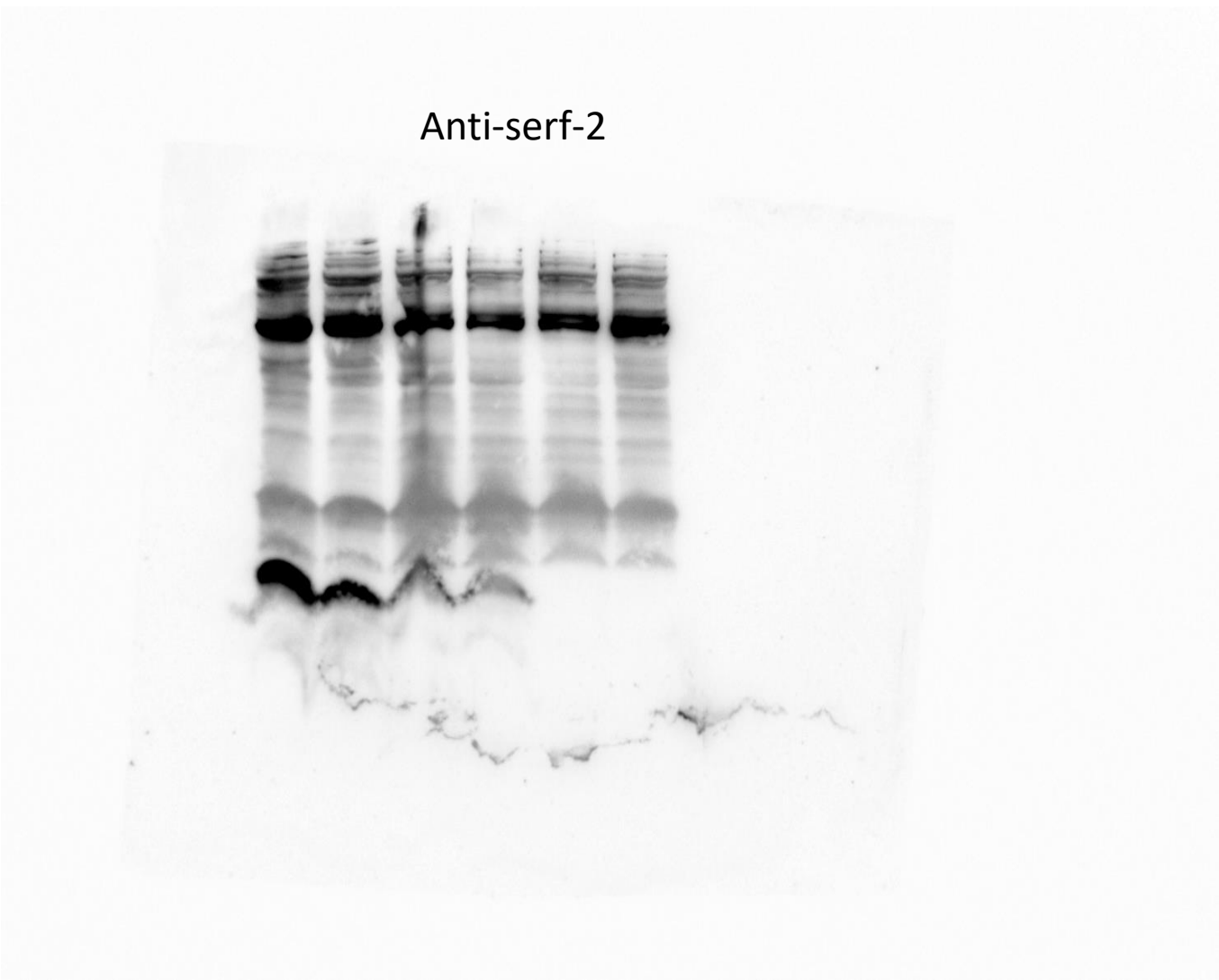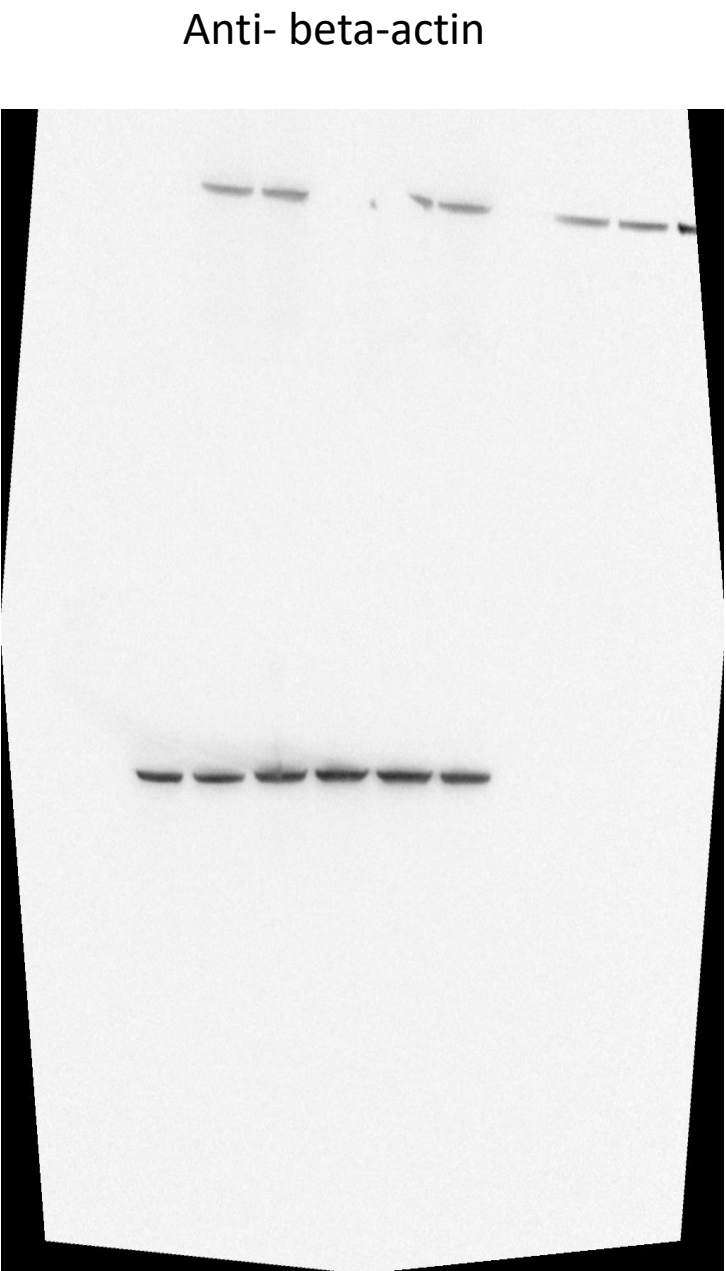

Figure 1F  
Wt

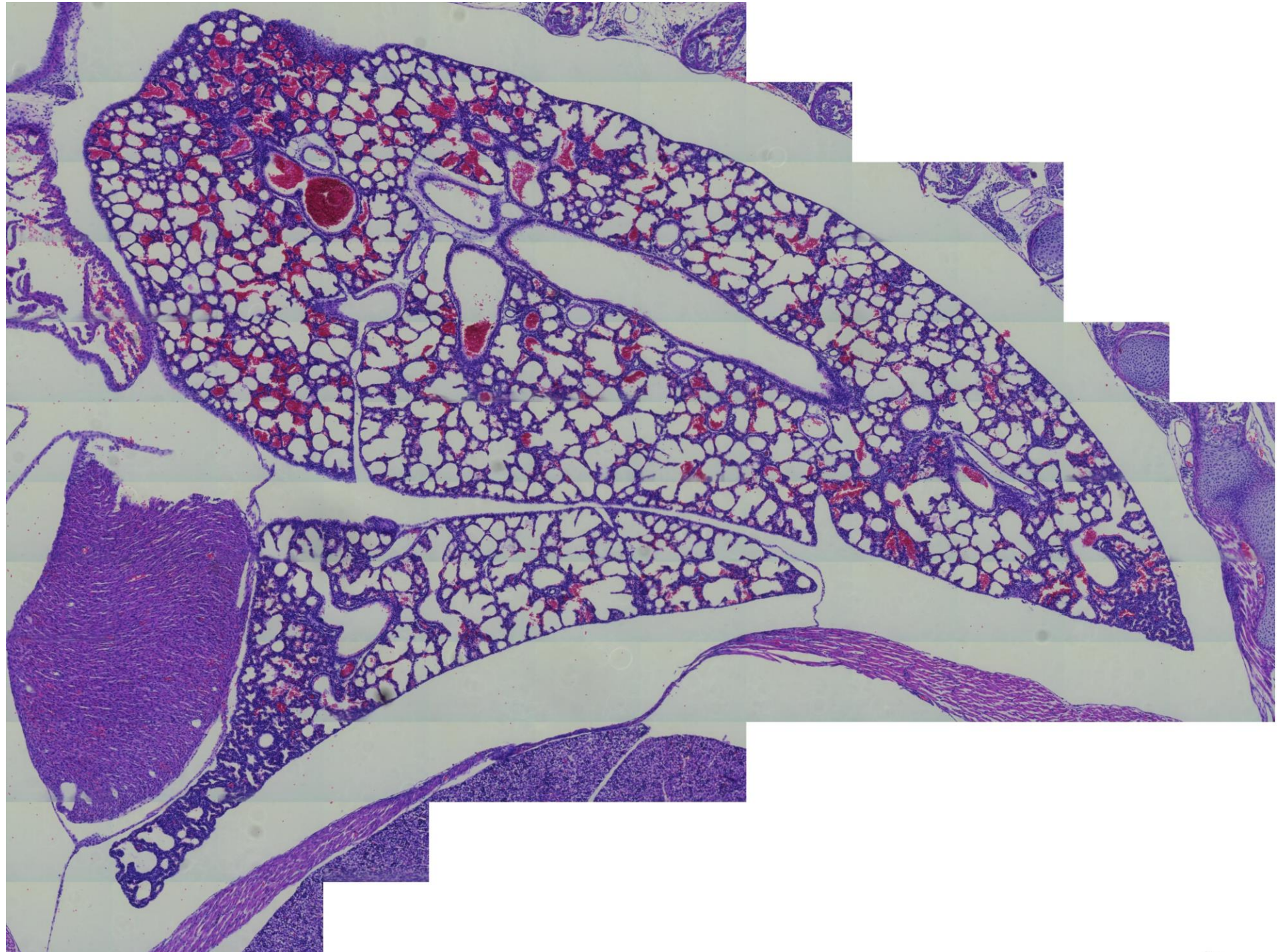

Figure 1F  
Serf -/-

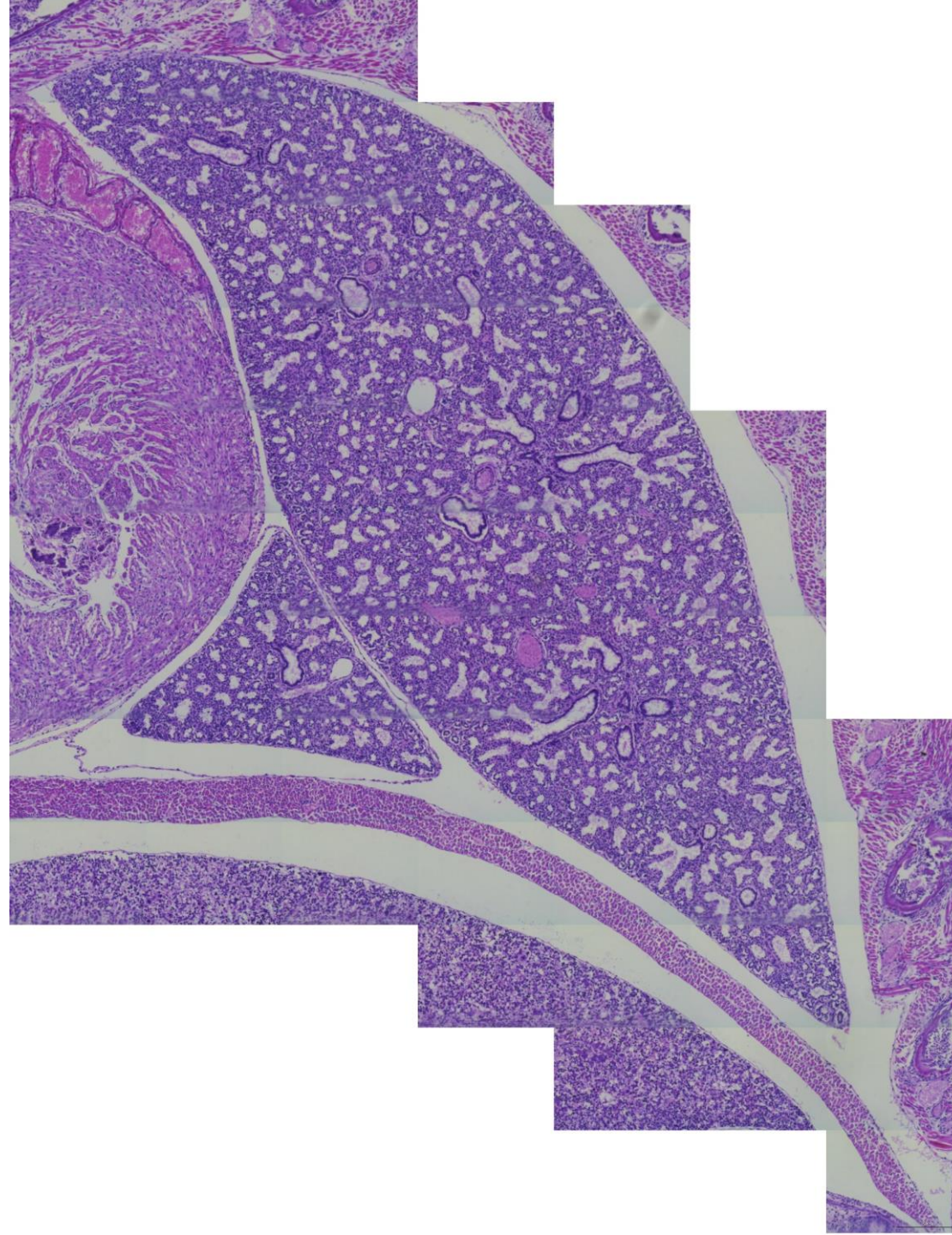

Supplement: Supplementary file 3 [file LSA-2022-01730_SdataF1.2.pdf]

AM

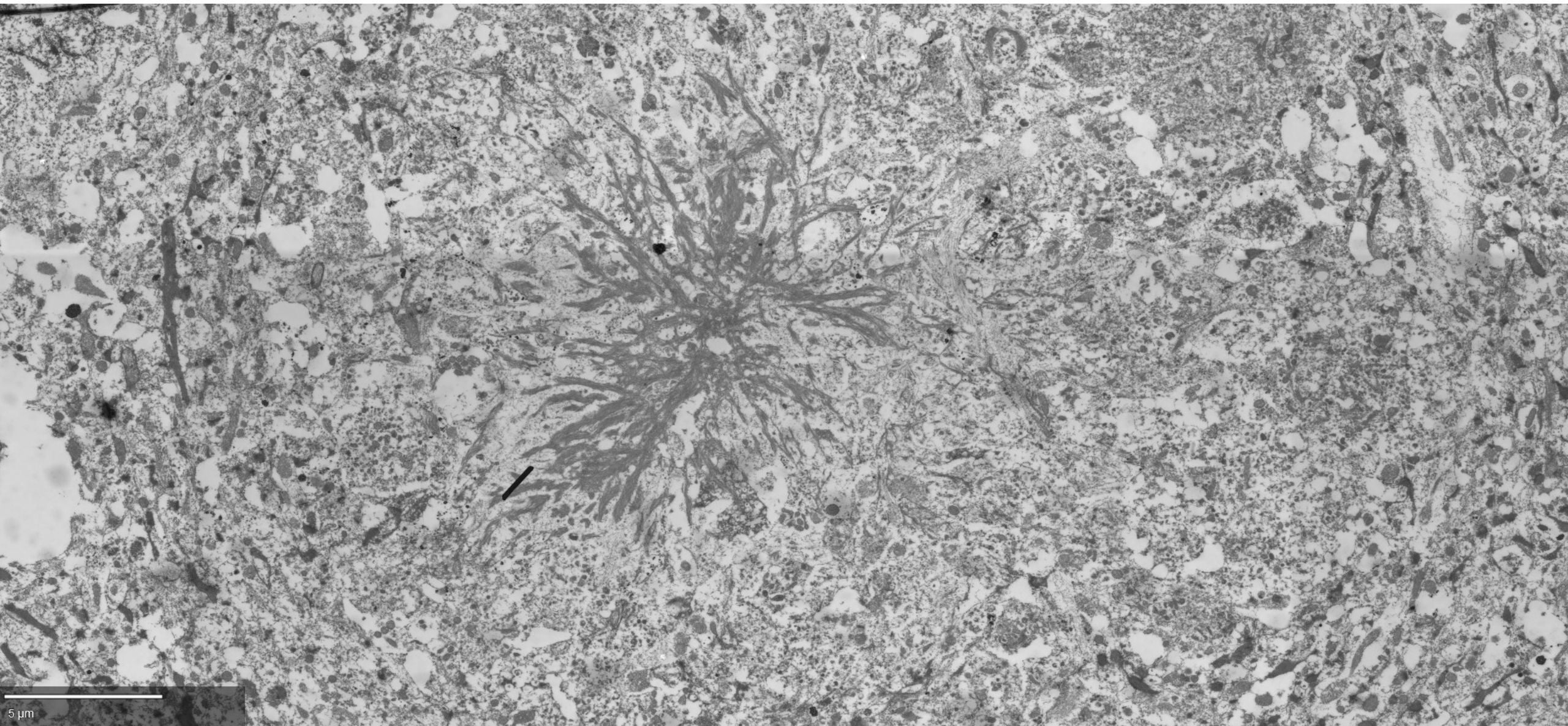

AM; serf -/-

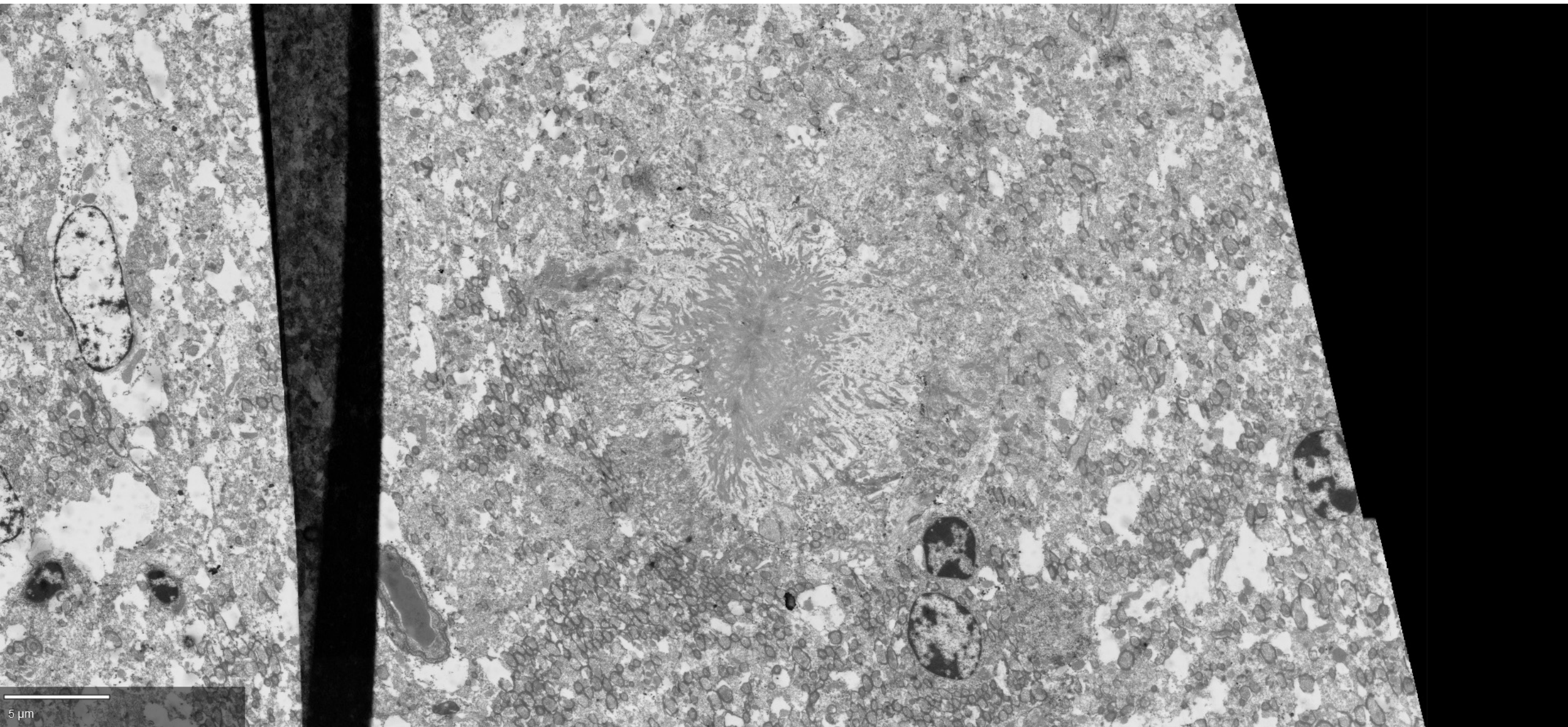

WT

KO

1

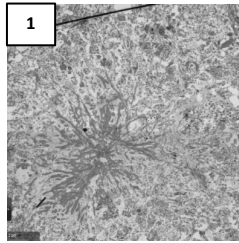

8

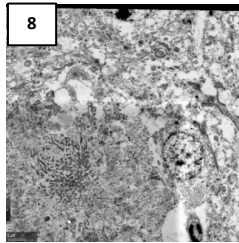

4

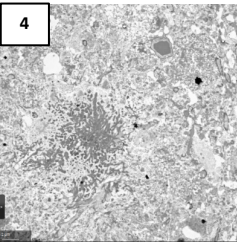

4

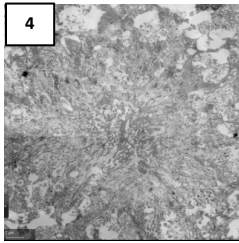

2

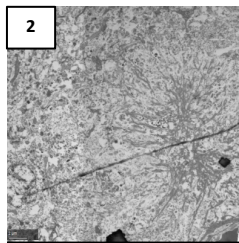

9

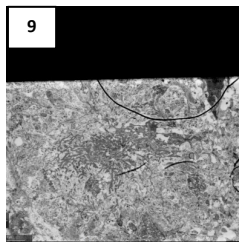

5

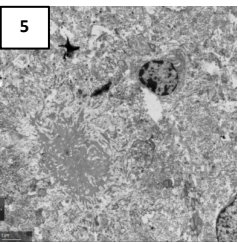

10

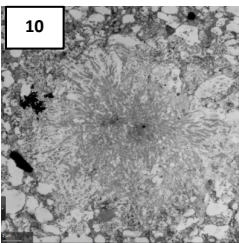

3

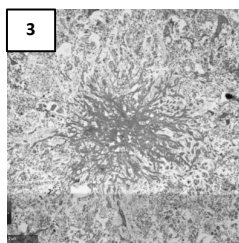

14

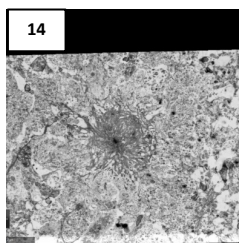

6

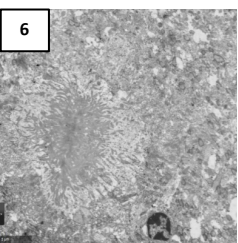

16

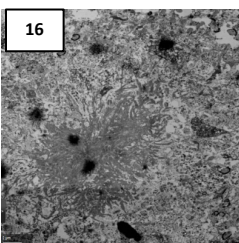

11

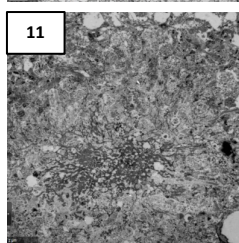

13

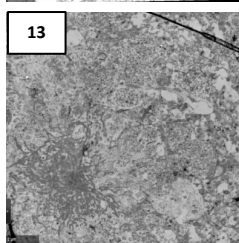

15

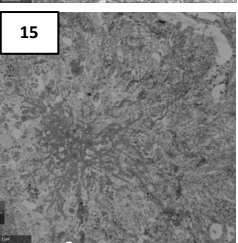

12

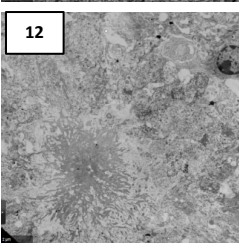

17

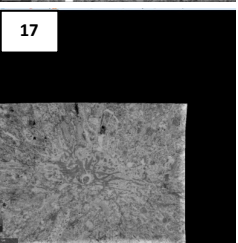

WT

KO

1

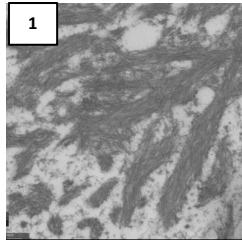

8

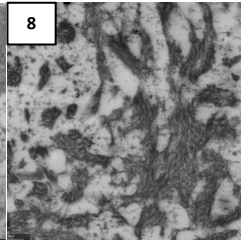

4

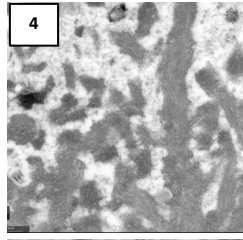

7

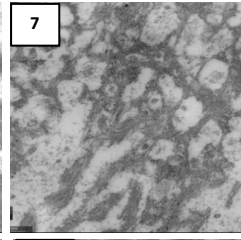

2

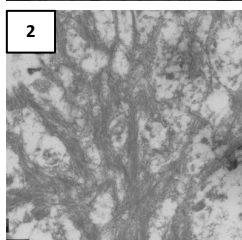

9

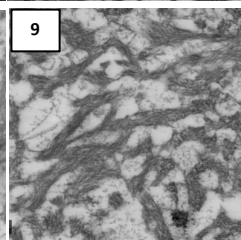

5

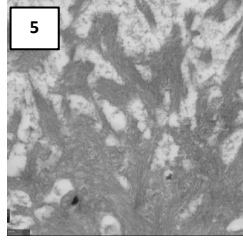

10

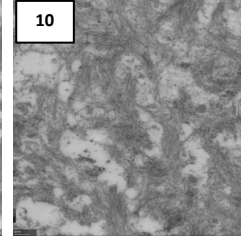

3

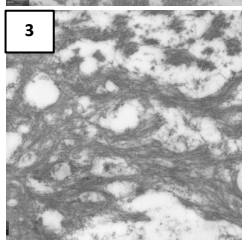

14

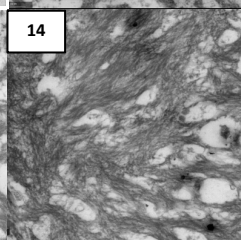

6

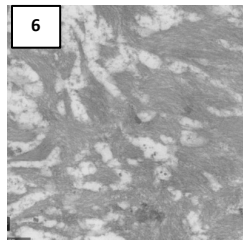

16

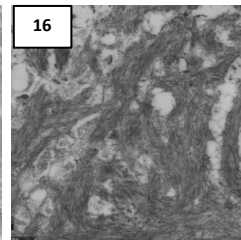

11

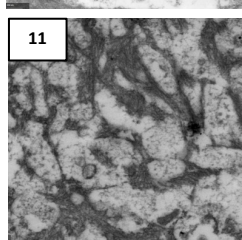

13

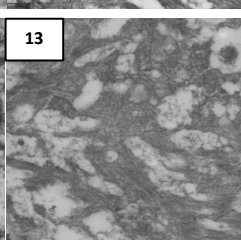

15

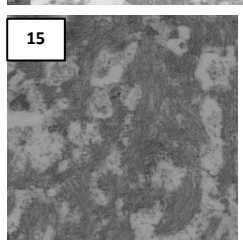

12

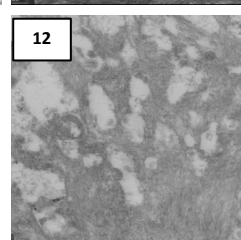

17

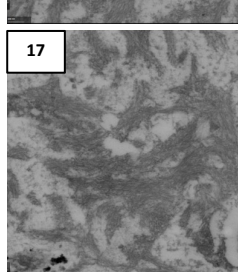

Supplement: Supplementary file 11 [file LSA-2022-01730_SdataF6.1.pdf]
