# Supplementary material for: Deletion of SERF2 in mice delays embryonic development and alters amyloid deposit structure in the brain
Source: Life Sci Alliance. 2023 May 2;6(7):e202201730. doi: 10.26508/lsa.202201730 (PMC10155860; doi:10.26508/lsa.202201730)
Supplement: Supplementary file 5 [file LSA-2022-01730_TableS2.docx]

**Table 2**: List of all differentially expressed genes in GO-term categories associated with the “Cell Cycle”-cluster from RNA sequencing analysis of Serf2 KO mouse embryonic fibroblasts (MEFs) compared to wild-type control MEFs.

| **Ensembl** | **Gene** | **logFC** | **FDR** |
| --- | --- | --- | --- |
| ENSMUSG00000030498 | Gas2 | -1,060126359 | 1,41698E-09 |
| ENSMUSG00000025408 | Ddit3 | -1,265215209 | 3,88374E-09 |
| ENSMUSG00000002068 | Ccne1 | 0,854350386 | 1,08815E-07 |
| ENSMUSG00000041324 | Inhba | 0,791251967 | 3,33503E-07 |
| ENSMUSG00000020205 | Phlda1 | 1,063176788 | 6,75472E-07 |
| ENSMUSG00000036390 | Gadd45a | -1,113212993 | 3,19009E-05 |
| ENSMUSG00000028678 | Kif2c | 0,597970691 | 0,000434739 |
| ENSMUSG00000020649 | Rrm2 | 0,678875434 | 0,000764196 |
| ENSMUSG00000049107 | Ntf3 | 0,899844231 | 0,000804724 |
| ENSMUSG00000024803 | Ankrd1 | 0,809921746 | 0,000829987 |
| ENSMUSG00000052957 | Gas1 | -0,760733546 | 0,000850053 |
| ENSMUSG00000072082 | Ccnf | 0,62576661 | 0,001058115 |
| ENSMUSG00000010067 | Rassf1 | 0,483034579 | 0,001074074 |
| ENSMUSG00000024912 | Fosl1 | 0,762241503 | 0,001169091 |
| ENSMUSG00000027342 | Pcna | 0,474065499 | 0,001284632 |
| ENSMUSG00000051220 | Ercc6l | 0,557913113 | 0,001376322 |
| ENSMUSG00000006398 | Cdc20 | 0,558763031 | 0,001451541 |
| ENSMUSG00000028212 | Ccne2 | 0,530677027 | 0,001548647 |
| ENSMUSG00000032487 | Ptgs2 | 0,764278479 | 0,001611556 |
| ENSMUSG00000030867 | Plk1 | 0,616889262 | 0,001706634 |
| ENSMUSG00000021253 | Tgfb3 | 0,464163337 | 0,002038909 |
| ENSMUSG00000069910 | Spdl1 | 0,609728329 | 0,002231623 |
| ENSMUSG00000019942 | Cdk1 | 0,57241497 | 0,002615369 |
| ENSMUSG00000039239 | Tgfb2 | 0,541006816 | 0,002802629 |
| ENSMUSG00000038668 | Lpar1 | -0,598647935 | 0,003114426 |
| ENSMUSG00000036777 | Anln | 0,537862661 | 0,003545435 |
| ENSMUSG00000001403 | Ube2c | 0,571370042 | 0,003585732 |
| ENSMUSG00000032171 | Pin1 | 0,441366932 | 0,003585732 |
| ENSMUSG00000029370 | Rassf6 | 0,846262635 | 0,004228902 |
| ENSMUSG00000028059 | Arhgef2 | -0,449448303 | 0,004470338 |
| ENSMUSG00000040093 | Bmf | -1,177186021 | 0,004719403 |
| ENSMUSG00000017716 | Birc5 | 0,524749041 | 0,005345195 |
| ENSMUSG00000021453 | Gadd45g | 0,506749122 | 0,006242552 |
| ENSMUSG00000040204 | 2810417H13Rik | 0,487521621 | 0,00720145 |
| ENSMUSG00000028873 | Cdca8 | 0,566216366 | 0,007804416 |
| ENSMUSG00000019214 | Chtf18 | 0,520978177 | 0,008415114 |
| ENSMUSG00000020897 | Aurkb | 0,52776024 | 0,008494094 |
| ENSMUSG00000028680 | Plk3 | 0,48580171 | 0,008705737 |
| ENSMUSG00000019923 | Zwint | 0,395094737 | 0,008705737 |
| ENSMUSG00000022070 | Bora | 0,627451257 | 0,008747691 |
| ENSMUSG00000023905 | Tnfrsf12a | 0,425100481 | 0,00903262 |
| ENSMUSG00000038943 | Prc1 | 0,486357249 | 0,009527625 |
| ENSMUSG00000026278 | Bok | 0,515693058 | 0,010470629 |
| ENSMUSG00000027654 | Fam83d | 0,470114843 | 0,01056321 |
| ENSMUSG00000030978 | Rrm1 | 0,45674763 | 0,011326467 |
| ENSMUSG00000041431 | Ccnb1 | 0,52578725 | 0,01150016 |
| ENSMUSG00000005410 | Mcm5 | 0,458598694 | 0,011760427 |
| ENSMUSG00000037544 | Dlgap5 | 0,465324614 | 0,013630161 |
| ENSMUSG00000025887 | Casp12 | -0,58915023 | 0,01378392 |
| ENSMUSG00000062380 | Tubb3 | 0,516648843 | 0,014601984 |
| ENSMUSG00000049932 | H2afx | 0,422478759 | 0,014826207 |
| ENSMUSG00000029363 | Rfc5 | 0,472471397 | 0,015366701 |
| ENSMUSG00000032254 | Kif23 | 0,473019921 | 0,016787926 |
| ENSMUSG00000020263 | Appl2 | -0,588286594 | 0,017171772 |
| ENSMUSG00000042489 | Clspn | 0,431195819 | 0,019791308 |
| ENSMUSG00000028851 | Nudc | 0,379712997 | 0,021729931 |
| ENSMUSG00000027715 | Ccna2 | 0,468421828 | 0,023070795 |
| ENSMUSG00000027996 | Sfrp2 | -0,783183522 | 0,023530353 |
| ENSMUSG00000028211 | Trp53inp1 | -0,459519649 | 0,023542054 |
| ENSMUSG00000000751 | Rpa1 | 0,351451213 | 0,023863918 |
| ENSMUSG00000026039 | Sgol2a | 0,402072781 | 0,023921416 |
| ENSMUSG00000029147 | Ppm1g | 0,349675652 | 0,025139066 |
| ENSMUSG00000012443 | Kif11 | 0,435593388 | 0,025289258 |
| ENSMUSG00000057789 | Bak1 | 0,36920948 | 0,025419654 |
| ENSMUSG00000001707 | Eef1e1 | 0,426204498 | 0,025943744 |
| ENSMUSG00000027859 | Ngf | 0,755023549 | 0,025964813 |
| ENSMUSG00000021469 | Msx2 | 0,691122321 | 0,026711446 |
| ENSMUSG00000029771 | Irf5 | 0,703930517 | 0,026765516 |
| ENSMUSG00000027496 | Aurka | 0,53380754 | 0,027247992 |
| ENSMUSG00000003235 | Eif2b5 | 0,333119079 | 0,027872662 |
| ENSMUSG00000041859 | Mcm3 | 0,415959378 | 0,029286611 |
| ENSMUSG00000017499 | Cdc6 | 0,416502924 | 0,029345645 |
| ENSMUSG00000027469 | Tpx2 | 0,391366488 | 0,029462682 |
| ENSMUSG00000001288 | Rarg | -0,438709791 | 0,029529703 |
| ENSMUSG00000023015 | Racgap1 | 0,447925189 | 0,029765523 |
| ENSMUSG00000002835 | Chaf1a | 0,444622315 | 0,029935382 |
| ENSMUSG00000001228 | Uhrf1 | 0,428632595 | 0,030132776 |
| ENSMUSG00000024521 | Pmaip1 | 0,756341726 | 0,03181244 |
| ENSMUSG00000025001 | Hells | 0,415757072 | 0,031840188 |
| ENSMUSG00000024989 | Cep55 | 0,510368968 | 0,03275137 |
| ENSMUSG00000025499 | Hras | 0,365294576 | 0,033224609 |
| ENSMUSG00000020647 | Ncoa1 | -0,512025385 | 0,034078322 |
| ENSMUSG00000024659 | Anxa1 | 0,414723092 | 0,034938798 |
| ENSMUSG00000026669 | Mcm10 | 0,517673204 | 0,03498066 |
| ENSMUSG00000027454 | Gins1 | 0,625748039 | 0,035319412 |
| ENSMUSG00000035439 | Haus8 | 0,398273944 | 0,035319412 |
| ENSMUSG00000034462 | Pkd2 | -0,337937806 | 0,036575157 |
| ENSMUSG00000028587 | Orc1 | 0,593702573 | 0,036773037 |
| ENSMUSG00000028896 | Rcc1 | 0,360227148 | 0,03907471 |
| ENSMUSG00000032477 | Cdc25a | 0,406071719 | 0,040949038 |
| ENSMUSG00000034165 | Ccnd3 | 0,332749823 | 0,044023388 |
| ENSMUSG00000022673 | Mcm4 | 0,363723782 | 0,044569436 |
| ENSMUSG00000020808 | Fam64a | 0,387435159 | 0,044773361 |
| ENSMUSG00000021483 | Cdk20 | -0,928473558 | 0,045110104 |
| ENSMUSG00000040152 | Thbs1 | 0,459754055 | 0,049416596 |
| Log FC values = logarithmic fold change of Serf2 KO vs control; genes highlighted in blue are downregulated in Serf2 KO | | | |
