# Supplementary material for: Deletion of SERF2 in mice delays embryonic development and alters amyloid deposit structure in the brain
Source: Life Sci Alliance. 2023 May 2;6(7):e202201730. doi: 10.26508/lsa.202201730 (PMC10155860; doi:10.26508/lsa.202201730)
Supplement: Supplementary file 7 [file LSA-2022-01730_TableS3.docx]

**Table 3**: Number of pups observed in the various genotype groups of the AD and AD;Serf2^br-/-^ cross.

| Genotype | Count |
| --- | --- |
| WT | 59 |
| Serf2^br-/-^ | 44 |
| AD | 60 |
| AD;Serf2^br-/-^ | 51 |
| Unknown | 21 |
| Total | **235** |
