# Supplementary material for: Deletion of SERF2 in mice delays embryonic development and alters amyloid deposit structure in the brain
Source: Life Sci Alliance. 2023 May 2;6(7):e202201730. doi: 10.26508/lsa.202201730 (PMC10155860; doi:10.26508/lsa.202201730)
Supplement: Supplementary file 10 [file LSA-2022-01730_TableS4.docx]

**Table 4**: Overview of main statistical parameters of linear regression analyses.

| X-component | Y-component | R^2^ AD | R^2^ AD;SERF2-/- | Slope (p-value) | Elevation (p-value) |
| --- | --- | --- | --- | --- | --- |
| Aβ42 | **Aβ40** | 0.8119 | 0.7887 | 0.5505 | 0.4257 |
| Aβ40 | **6E10 deposits** | 0.8159 | 0.9185 | 0.7798 | 0.0040** |
| Aβ42 | **6E10 deposits** | 0.8701 | 0.7774 | 0.4835 | 0.0470* |
| Aβ40 | **ThS deposits** | 0.6553 | 0.5572 | 0.0075** | NA |
| Aβ42 | **ThS deposits** | 0.6295 | 0.3690 | 0.0150* | NA |
| 6E10 deposits | **W0-2 deposits** | 0.5508 | 0.8982 | 0.1443 | 0.7724 |
| 6E10 deposits | **ThS deposits** | 0.5974 | 0.0099 | 0.0932 | 0.5157 |
| 6E10 deposits | **Intensity Ratio** | 0.0856 | 0.4377 | 0.4726 | 0.0107* |
| W0-2 deposits | **Intensity Ratio** | 0.0006 | 0.3351 | 0.4369 | 0.0202* |
| ThS deposits | **Intensity Ratio** | 0.0064 | 0.2595 | 0.3716 | 0.0376* |
