# Supplementary material for: Deletion of SERF2 in mice delays embryonic development and alters amyloid deposit structure in the brain
Source: Life Sci Alliance. 2023 May 2;6(7):e202201730. doi: 10.26508/lsa.202201730 (PMC10155860; doi:10.26508/lsa.202201730)
Supplement: Supplementary file 13 [file LSA-2022-01730_TableS5.docx]

**Table 5:** List of used primers.

| Application | Gene | Forward | Reverse |
| --- | --- | --- | --- |
| qPCR | Mouse Serf2 | CCGCGGTAACCAGCGAGAGC | TCCGAGTCCCTCTGCTTGCG |
| qPCR | Mouse Serf1 | TGGCCCGTGGAAATCAAAGAGAAA | TGCATGATCTCTGAATCCCTCTGCT |
| qPCR | Human APP | CAGAATTCCGACATGACTCAGGATATGAAG | CCCACCATGAGTCCAATGATTGC |
| qPCR | Mouse 18S | CGGACAGGATTGACAGATTG | CAAATCGCTCCACCAACTAA |
| qPCR | Mouse β-ACTIN | CTCCTCCTGAGCGCAAGTACTCTGTGT | GTGCACGATGGAGGGGCCGGACTCAT |
| Genotyping | Cre | AGCCTGTTTTGCACGTTCACC | GGTTTCCCGCAGAACCTGAA |
| Genotyping | Serf2 flox | ATGACCCGTGAGTGCTGGGAC | GACGGTGCGGTCGAGACTGC |
| Genotyping | AD transgenes (APP) | GTCATAGCGACAGTGATCGT | ACTTAGGCAAGAGAAGCAGC |
| Genotyping | AD transgenes (PS1) | CAGGTGCTATAAGGTCATCC | ATCACAGCCAAGATGAGCCA |
