# Supplementary material for: Deletion of SERF2 in mice delays embryonic development and alters amyloid deposit structure in the brain
Source: Life Sci Alliance. 2023 May 2;6(7):e202201730. doi: 10.26508/lsa.202201730 (PMC10155860; doi:10.26508/lsa.202201730)
Supplement: Supplementary file 14 [file LSA-2022-01730_TableS6.docx]

**Table 6:** List of antibodies used for Western blotting and immunostaining.

| Antigen | Host | Manufacturer | Cat # | Dilution |
| --- | --- | --- | --- | --- |
| Serf2 | Rabbit | Protein Tech | 11691-1-AP | 1/1500 |
| 6E10 (Aβ 1-16) | Mouse | Covance | 803002 | 1/1000 |
| W02 (Aβ 4-10) | Mouse | Millipore | MABN10 | 1/1000 |
| α-Actin | Mouse | MP Biomedicals | 691001 | 1/100,000 |
